# Incidence of Endophthalmitis after Intravitreal Anti-Vascular Endothelial Growth Factor Injections in an Operating Room in China

**DOI:** 10.1155/2020/5163484

**Published:** 2020-11-12

**Authors:** Yanyun Chen, Wenbin Wei, Demetrios G. Vavvas, Feng Zhang, Haicheng She, Haiying Zhou, Lei Li, Yao Huang, Dimitrios P. Ntentakis, Xiangyu Shi

**Affiliations:** ^1^Beijing Tongren Eye Center, Beijing Key Laboratory of Ophthalmology and Visual Science, Beijing Tongren Hospital, Capital Medical University, Beijing, China; ^2^Department of Ophthalmology, Massachusetts Eye and Ear Infirmary, Harvard Medical School, Boston, MA, USA

## Abstract

**Purpose:**

To evaluate the rate of presumed endophthalmitis (EO) after intravitreal anti-vascular endothelial growth factor (anti-VEGF) injections performed in an operating room (OR) under sterile conditions in mainland China.

**Methods:**

Retrospective single-center study between September 2012 and December 2017 at Beijing Tongren Eye Center, Beijing, China. Intravitreal injection database was reviewed. All anti-VEGF injections were performed using a standardized sterile technique in an OR. Injection protocols included antibiotics for 3 days pre-injection, topical 5% povidone-iodine rinsing before the procedure, and post-injection antibiotics for 3 days.

**Results:**

A total of 37,830 intravitreal injections were performed at Beijing Tongren Eye Center. Three cases were managed as presumed EO (0.0079%). Positive cultures were documented in 2 of 3 cases. EO incidence following ranibizumab and conbercept administration was 0.0088% (3 in 33,930) and 0% (0 in 3,900), respectively. No significant difference was detected between the two drugs (*P* = 0.745).

**Conclusions:**

Very low EO rates were seen in mainland China using a standardized sterile technique in an OR. However, EO could not be completely avoided.

## 1. Introduction

Intravitreal anti-vascular endothelial growth factor (anti-VEGF) injections are considered the standard treatment for patients with neovascular age-related macular degeneration (AMD), diabetic retinopathy (DR), and macular edema secondary to retinal vein occlusion. However, the significant therapeutic benefits over the years have not been completely free of complications. Following any intravitreal injection, there is always a risk of non-infectious or infectious endophthalmitis (EO). Non-infectious EO refers to a transient, self-limited inflammatory reaction, typically with hypopyon, presenting within the first few days after injections [1]. Infectious EO is overall the most feared complication of intravitreal injections. Typical bacterial EO presents with rapid visual loss, eye pain, conjunctival hyperemia, hypopyon, and vitreous opacification [2]. Bacterial infections account for the majority of such cases [3].

Variations in post-injection EO incidence might be explained by the different injection protocols, procedure settings (operating room (OR) versus office-based), and prophylactic antibiotics. In a retrospective multicenter study in Europe, EO incidence was reported lower (1/13,470) because anti-VEGF injections were administered in the higher sterility environment of an OR [4]. Meanwhile, intravitreal injections performed in the office setting achieved a comparatively low EO rate (0.009%) in a retrospective review of 10,142 consecutive cases [5]. Topical antibiotic prophylaxis is still controversial [6, 7], but more and more studies report low EO incidence without prophylaxis [8, 9]. In one study, the EO rate in the group with topical antibiotic prophylaxis after intravitreal injection was similar to that in the group without prophylaxis [8]. Using conjunctival sac irrigation with 0.25% povidone-iodine (PVI) before and after intravitreal injection, the EO incidence (0/12,523, 95% CI 0–0.00024%) remained low without pre- or post-injection topical antibiotics in a Japanese study [9].

Over the last decade, anti-VEGF injection demand has exponentially increased in China. In compliance with the 2015 Chinese Ocular Fundus Society (COFS) guidelines, intravitreal injections are performed in an OR, using 5% PVI irrigation before the injection, and topical antibiotics pre- and post-injection [10]. Compared to other countries [5, 9], the injection setting and the use of prophylactic antibiotics might be slightly different in China. Nonetheless, no report of post-injection EO has been documented to date.

This study presents post-injection EO rates from the largest eye center in mainland China, while co-evaluating the most frequent pathogens, visual outcomes, and potential risk factors.

## 2. Subjects and Methods

### 2.1. Subjects

All patients who had received intravitreal anti-VEGF injections with ranibizumab (Lucentis; Novartis AG, Basel, Switzerland) and conbercept (Lumitin; Kanghong Biotech, Chengdu, PR China) between September 2012 and December 2017 were enrolled. The injections were performed at the Beijing Tongren Eye Center, Beijing, China. Presumed EO was defined as any intraocular inflammation treated with tap and inject, with or without vitrectomy. Aqueous humor and vitreous samples were cultured. All presumed EO cases were included regardless of the subsequent culture results, positive or negative. EO cases were excluded from our study if the intravitreal injection had been combined with another ocular procedure (i.e., cataract operation, vitrectomy, laser, and paracentesis). Informed consent was obtained from all patients before the injections. Our study was conducted in accordance with the tenets of the Declaration of Helsinki and approved by the Ethics Committee of the Beijing Tongren Hospital, Capital Medical University. The minimum follow-up duration for all cases in this study was one year.

### 2.2. Intravitreal Injection

Our procedures complied with the 2015 consensus of the COFS [10]. All intravitreal injections were performed in the OR without laminar airflow. All physicians wore disposable caps and surgical masks. Hand and arm disinfection was followed by the use of sterile disposable surgical gowns. Disposable clothing and surgical caps were worn by the patients as well. Additional sterilization measures included local antiseptics, sterile drapes, and sterile gloves. Disposable injection sets and all necessary instruments were prepared on a sterile tray. Peri-ocular scrub with 10% PVI was performed three times per case before the injection. An adhesive surgical drape and a sterile eyelid speculum were used. All injections were preceded by 5% PVI rinsing of the fornices for at least 90 seconds, which was then washed out twice using 0.9% normal saline. A 30-gauge (G) needle was used for the procedure while avoiding any contact with the eyelids and the speculum. Topographically, the entry site was set at 3.5 mm posterior to the limbus in pseudophakic/aphakic eyes and at 4.0 mm from the limbus in phakic eyes. After the injection, antibiotic ointment (tobramycin and dexamethasone ophthalmic ointment) was applied on the lid conjunctiva, and the periorbital region was gently cleaned using sterile compresses. All patients received levofloxacin eye drops four times per day for 3 days pre-injection and 3 days post-injection.

### 2.3. Statistical Analysis

Confidence intervals were calculated by using the Wilson score method without continuity correction. The confidence limits for the relative risk reduction are one minus the confidence limits for the relative risk. Fisher's exact probability method was used to compare the EO incidence between ranibizumab and conbercept groups.

## 3. Results

A total of 37,830 intravitreal anti-VEGF injections were performed at Beijing Tongren Eye Center from September 2012 to December 2017. Three cases of presumed EO were documented in 33,930 (ranibizumab, 95% CI: −0.0012% to 0.0188%) and 0 in 3,900 (conbercept). A combined incidence per injection is 0.0079% (1/12,610). There was no significant difference between the two drugs (*P* = 0.745).

EO cases were presented in June 2014 (Case 1), June 2015 (Case 2), and May 2017 (Case 3), respectively. All 3 presumed EO cases were initially managed with tap and inject at the emergency OR. After 24 hours, each case was evaluated based on visual acuity (VA) measurements, as well as the presence of eye pain, redness, and vitreous opacification. In all 3 cases, the aforementioned clinical signs persisted and rapidly worsened, accompanied by declining visual acuity during follow-up. Thus, pars plana vitrectomy (PPV) was immediately performed on follow-up day 1. Positive cultures were drawn from 2 of 3 cases, revealing *Staphylococcus epidermidis* and *Streptococcus pneumonia*, respectively. Each patient's ophthalmologic history, demographics, intraocular pathogen, and associated comorbidities such as high myopia, diabetes mellitus, and AMD are summarized in Table 1.

## 4. Discussion

Intravitreal injection of anti-VEGF has revolutionized the management and visual prognosis for patients with AMD, diabetic macular edema, and other diseases, resulting in retinal vascular leakage or choroidal neovascularization. Each such procedure is independently associated with a small risk of EO, ranging from 1 in 2,000 to 1 in 10,000 [4, 11–13]. EO post-injection may represent about 8.5% of the total EO cases [14].

The number of patients receiving intravitreal injections is continuously increasing. As procedural changes have been continuously evolving to reduce injection-related injury and infection, low EO rates can be achieved [15–17]. Our study has similarities to the retrospective multicenter study from the three European sites where 134,701 intravitreal injections were performed via a standardized sterile technique in the OR setting. The reported EO rate was 0.0074% per injection (40% culture positive) [4]. In contrast to our study, laminar flow was used in the OR, no pre-injection antibiotics were given, and postoperative antibiotics varied among the different sites [4]. In our study, we found a similar combined incidence per injection (0.0079% or 1/12,610) with 2 out of 3 positive cultures (one case of *Staph. epidermidis* and one of *Strept. pneumonia*). Another large sample of intravitreal injections performed in a positive pressure ventilated operating room under sterile conditions in Denmark also showed very low incidence of EO (zero in 20,293 injections) [18]. The physicians wore face mask, the conjunctiva was irrigated twice with 5% PVI preoperatively, and topical tobramycin was applied immediately after the injection. Post-injection antibiotics were given for all patients during the first 5 years of the study and then, only to patients with diabetes [18]. On the basis of such outcomes, one may advocate a standardized, sterile, OR-based technique as a means to reduce EO risk, regardless of the use of laminar flow. However, office-based intravitreal injections in three large studies showed a comparatively low EO rate (0.009%, 0.02%, and 0.029%) without the use of an OR [5, 19, 20]. So far, there is no prospective case-control study comparing EO incidence for intravitreal injections performed in an outpatient setting versus an OR. As intravitreal injections have significantly increased over the past few years, an office-based setting might not only be more efficient but also more affordable and comparably safe.

Since there is always a risk of infectious or non-infectious endophthalmitis following any intravitreal injection, we should be vigilant to eliminate confounders. In general, eyes with marked fibrin, severe eye pain, and profound vision loss are more likely to be infectious EO. In contrast, eyes with minimal or absent fibrin, no pain or mild pain, self-limited, and better vision are probably non-infectious EO [1]. Non-infectious postinjection EO rates vary amongst studies [21]. Cases tended to cluster instead of occurring at a consistent rate every year. Yamashiro et al. reported that 14 eyes developed sterile endophthalmitis post-injection from a single batch [22]. Similarly, Entezari et al. reported 11 eyes developed sterile endophthalmitis after intravitreal bevacizumab injections from the same batch [23]. Pain, redness, and decreased vision began 11–17 days post-injection, yet prognosis was favorable for all cases eventually. On the other hand, non-infectious EO incidence may vary with different drugs. For instance, non-infectious EO incidence was higher for bevacizumab compared with ranibizumab and aflibercept [24]. In our study, all 3 EO cases progressed very rapidly with severe eye pain, worsening conjunctival hyperemia, and vitreous body opacification. Even though 1 case was culture-negative, it was also regarded as infectious EO due to its clinical manifestations.

In our study, the underlying diseases of the 3 presumed EO cases were pathologic myopia, diabetic retinopathy, AMD with entropion, and blepharitis. Scleral thinning secondary to high myopia, increased predisposition to infection with diabetes and as well as the propensity of conjunctivitis due to entropion and blepharitis are well-known EO risk factors. The patient with pathologic myopia also reported washing her hair within less than 24 h after the injection. Multiple injections and myopia-related scleral thinning might also increase the risk of bacteria invasion through the thinner scleral tunnel into the vitreous body. Since diabetics have an increased prevalence of infections [25], some studies have suggested that endophthalmitis following invasive ophthalmic procedures such as cataract surgery or PPV may occur more frequently in patients with diabetes [26, 27]. Post-injection EO rates have been reported higher in eyes with diabetes (0.049%) compared with eyes with retinal vein occlusion (0.012%, *P* = 0.010) [28]. In a retrospective study, 8 EO cases occurred among 15,925 intravitreal injection. Of these, 3 cases (3/8) had a history of diabetes [29]. Although culture-negative, one of the three presumed EO cases in our study was also diabetic. Given the well-known susceptibility of diabetics to prolonged asymptomatic infections, we suggest that more attention should be paid to all such patients requiring intravitreal injections.

There was no difference in EO rates between different anti-VEGF drugs (*P* = 0.745) in our study. Our results agree with previous reports in this field. In one of them, the EO incidence using ranibizumab, aflibercept, and bevacizumab was 0.020%, 0.021%, and 0.020%, respectively, with no significant difference between the drugs (*P* = 0.896) [24]. In a multicenter retrospective cohort study, 183 presumed EO cases were identified from a total of 503,890 anti-VEGF injections [30]. EO rate following intravitreal bevacizumab (0.039%, 60/153,812), ranibizumab (0.035%, 109/309,722), and aflibercept (0.035%, 14/40,356) injection again appeared similar [30]. Therefore, we suggest that the choice of anti-VEGF agents should be primarily based on their individualized clinical efficacy rather than the potential risk of infection.

Despite its low mathematical value, EO risk independently increases for any patient receiving a new intravitreal injection. 5% PVI effectively disrupts conjunctival bacterial colonies, and 30 seconds of exposure appears to be an adequate time to decrease conjunctival bacterial counts [31]. PVI has the advantage of not invoking drug resistance, unlike antibiotics [32]. The use of pre- and post-injection topical antibiotics for prophylaxis has been debated [12, 33–36]. Widespread use of prophylactic antibiotics may underline the alarming rates of antibacterial treatment failure and antibiotic resistance. In a retrospective case-control study of 172,096 anti-VEGF injections [33], 28 EO cases (10 culture-positive) occurred among 57,654 injections with post-injection antibiotic prophylaxis versus 24 EO cases (6 culture-positive) among 89,825 injections without antibiotics. In the post-injection antibiotics use cohort, four out of ten (40%) culture-positive cases grew bacteria resistant to the prescribed prophylactic antibiotics. In contrast, none of the six culture-positive cases in the cohort without post-injection antibiotics use grew resistant bacteria (odds ratio = 9.0; 95% confidence interval = 0.40–203.3; *P* = 0.17). This study suggested that prophylactic topical antibiotics following intravitreal injection may lead to higher rates of antibiotic-resistant bacteria in culture-positive EO cases [33]. A recent systematic review further advocates that antibiotic prophylaxis does not reduce the post-injection EO rate, while it might potentially be associated with an increased risk of post-operative infection [7]. Similarly, EO incidence might not increase without antibiotics. In one study, all non-diabetic patients (approximately 7,000 injections) were treated without post-injection topical antibiotics and no one suffered from EO [18]. Thus, omitting topical antibiotics might be safe in patients without diabetes. In our study, all patients received levofloxacin eye drops four times per day for 3 days pre-injection and 3 days post-injection. However, our EO incidence was not lower than other reports. Our results further advocate that overall EO rates do not decrease even with prophylactic antibiotics. In 2015, American Society of Retina Specialists (ASRS) Annual Preferences and Trends (PAT) survey reported an ongoing divergence between US and international members about topical antibiotics use in intravitreal injections: 9.5% of US members reported using topical antibiotics, as opposed to 60.6% of international members [37]. In 2016, the PAT survey showed only 8.8% of US members and 53.5% of international members reported use of topical antibiotics [38]. The PAT survey reveals a decline in topical antibiotics over the years. Based on many newer published series of intravitreal injections without topical antibiotics [8, 9], it is possible that non-US ophthalmologists will increasingly forgo topical antibiotics with intravitreal injections.

Regardless of the injection setting and antibiotic prophylaxis, prefilled syringes as well as the large-gauge needles used in long-term release devices might also affect EO incidence [39–41]. In our study, ranibizumab and conbercept were packaged in a glass vial and aspirated with a large bore needle before injection. None of prefilled syringes were used in this study.

The current study has several limitations. First, it is a single-center retrospective study. Second, the OR without laminar airflow might affect EO incidence. Third, with the combination of prophylactic topical antibiotics and irrigation with 5% PVI rinsing before the procedure, we cannot distinguish the role of antibiotics versus 5% PVI alone. Finally, the intravitreal injection in this study is OR-based. An OR setting not only increases the cost but also prolongs waiting hours for the patients. Combined with the rapidly increasing demand for intravitreal injections in China, an outpatient setting should be explored in an attempt to enhance efficiency and cost-effectiveness. To date, though we have no data for intravitreal injections performed in a clinical office. From our standpoint, a prospective study monitoring post-injection EO rates in a standardized outpatient setting in China would be extremely beneficial for the optimization of healthcare practices in the country.

## 5. Conclusions

In conclusion, a standardized sterile technique in an OR showed very low EO rates at mainland China. However, EO could not be completely avoided.

## Figures and Tables

**Table 1:** Key data for the patients with endophthalmitis.

	BCVA								
	Age	Gender	Injection numbers	Onset (days)	Prior	At Dx of EO	Final	Culture results	Other notes:
Case 1	23	F	7	2	0.6	HM	0.3	*Staphylococcus epidermidis*	Myopic CNV, washing hair on the day of injection
Case 2	60	M	1	2	HM	HM	FC	Negative	Diabetic retinopathy, diabetes mellitus
Case 3	67	M	1	4	LP	LP	FC	*Streptococcus pneumonia*	AMD, entropion, and blepharitis

M: male; F: female; BCVA: best-corrected visual acuity; Dx: diagnosis; HM: hand movement; CNV: choroidal neovascularization; FC: finger counting; LP: light perception.

## Data Availability

The data used to support the findings of this study are available from the corresponding author upon request.
